# An Ensemble Filter for Indoor Positioning in a Retail Store Using Bluetooth Low Energy Beacons

**DOI:** 10.3390/s19204550

**Published:** 2019-10-19

**Authors:** Vasilis Stavrou, Cleopatra Bardaki, Dimitris Papakyriakopoulos, Katerina Pramatari

**Affiliations:** 1 Department of Management Science and Technology, Athens University of Economics and Business, 76 Patission Str., 10434 Athens, Greece; k.pramatari@aueb.gr; 2 Department of Informatics and Telematics, Harokopio University of Athens, 9 Omirou Str., 17778 Athens, Greece; cleobar@hua.gr; 3 Department of Business Administration, University of West Attica, Campus 2, 250 Thivon & P. Ralli, 12241 Athens, Greece; dpapak@uniwa.gr

**Keywords:** Bluetooth Low Energy, indoor positioning, BLE Beacons, ensemble filter, fingerprinting, retail store

## Abstract

This paper has developed and deployed a Bluetooth Low Energy (BLE) beacon-based indoor positioning system in a two-floor retail store. The ultimate purpose of this study was to compare the different indoor positioning techniques towards achieving efficient position determination of moving customers in the retail store. The innovation of this research lies in its context (the retail store) and the fact that this is not a laboratory, controlled experiment. Retail stores are challenging environments with multiple sources of noise (e.g., shoppers’ moving) that impede indoor localization. To the best of the authors’ knowledge, this is the first work concerning indoor localization of consumers in a real retail store. This study proposes an ensemble filter with lower absolute mean and root mean squared errors than the random forest. Moreover, the localization error is approximately 2 m, while for the random forest, it is 2.5 m. In retail environments, even a 0.5 m deviation is significant because consumers may be positioned in front of different store shelves and, thus, different product categories. The more accurate the consumer localization, the more accurate and rich insights on the customers’ shopping behavior. Consequently, retailers can offer more effective customer location-based services (e.g., personalized offers) and, overall, better consumer localization can improve decision making in retailing.

## 1. Introduction

The advent of location-based technologies and the explosive growth of positioning equipment, such as global positioning system (GPS) receivers, radio frequency identification (RFID), Wi-Fi and Bluetooth, have facilitated tracking several objects moving through indoor or outdoor environments [1]. At the same time, the continuous evolution and usage of consumer electronics, such as mobile phones, highlights a new infrastructure for location-based services, which are getting more and more attention by academics and practitioners alike [2]. Location-based services identify the location of an object (e.g., a person, a car, or a shopping cart) and utilize it to provide useful information and content to the users of the service. These location-based services are applied to numerous fields and foster new use-cases. For instance, they are used to provide navigation in shopping malls or public buildings, to offer museum guidance, to ease product localization in supermarkets and indoor advertisements [3].

Thus, accurate object/human positioning is necessary for providing effective location-based services. Although it is not so difficult and complex to locate the exact position of an object moving outdoors (e.g., using GPS signals and geofencing techniques [4]), this becomes a quite challenging task in indoor environments. Indeed, the indoor positioning issue appears in the relevant literature as a very challenging one and the achievement of high localization precision (accuracy) is a common objective shared by various scholars [5,6]. Lymberopoulos et al. [7] argued that the indoor location problem still remains unsolved and stresses the importance of the employment of a realistic approach that would counterbalance the desired localization accuracy with low costs. Most studies have utilized Wi-Fi technology for indoor localization experimenting with a vast variety of indoor positioning techniques (e.g., [8,9,10,11]), while research on Bluetooth technology for indoor positioning is increasing (e.g., [4,12,13,14,15]). However, most works are practically laboratory experiments (e.g., [16,17,18,19,20]) that have not validated their indoor positioning approach in a real, fully operational environment.

This paper has developed a low-cost Bluetooth Low Energy (BLE) beacon-based indoor positioning system and has put it into practice in a real grocery retail store with two floors of different layout characteristics for shoppers’ positioning during their shopping trips. The ultimate purpose of this study was to apply and compare different indoor positioning techniques towards achieving the most possible efficient position determination of moving customers in the retail store. The key innovation of this research lies in the context of this research (i.e., the retail store) and the fact that this is not a laboratory experiment, namely a controlled context.

Retail stores are challenging environments that change often in terms of layout, product variety etc. Moreover, there are multiple sources of noise in the stores (e.g., shoppers’ and staff moving, products, mobile devices) that make indoor localization a challenging task. To the best of the authors’ knowledge, this is the first work concerning indoor localization of consumers in a fully operational retail store.

Since indoor localization in retail environments is still unexplored, this study started by assessing the performance of the most common techniques for indoor positioning (i.e., trilateration and fingerprinting) and established classifiers. It was found that the random forest is the best classifier. However, this study moved on to proposing and assessing an ensemble filter. The absolute mean and root mean squared errors of the ensemble filter are significantly lower (40.7% and 18% lower, respectively). More specifically, for approximately 70% of our cases (captured events of consumers), the ensemble method results in a localization error of less than 1 m and in 80% of the cases, the localization error is approximately 2 m. On the contrary, for the random forest, in 80% of the cases, the localization error is approximately 2.5 m. 

In retail environments in particular, such a deviation is significant, because even 0.5 m away from the actual shopper’s position may lead to position him in a different shopping isle and in front of a different store shelve, thus a different product category. Namely, the more accurate localization of consumers, the more accurate and rich insights on the customers’ shopping behavior. Consequently, the retailers and the marketing managers will be able to offer more effective customer location-based services (e.g., personalized offers, coupons etc. reflecting their recent shopping trips). Overall, the authors propose that it is worth moving forward from the random forest and proposing an ensemble method because better consumer localization results in better decision making in the retail industry.

Next, in Section 2, the existing literature of indoor positioning techniques and technologies are briefly reviewed, emphasizing on works that have applied Bluetooth technology and discussing the gaps that this work attempts to address. Section 3 presents the architecture of our BLE Beacon based indoor positioning system and Section 4 describes how it was deployed in the retail store. Section 5 presents the evaluation study of the indoor positioning techniques in the retail store, as well as the authors’ proposed hybrid approach for determining the shoppers’ positions. In Section 6, the results and the practical implications are briefly discussed, and in Section 7, the research limitations and plans for further research are discussed.

## 2. Related Work

The popularization of location-based technologies, such as Wi-Fi, Bluetooth and radio-frequency identification (RFID), has facilitated users to track and observe several objects moving into indoor environments [1,21]. At the same time, massive amounts of data are generated while these objects move around in various environments. However, several challenges may arise. A common challenge regards how these data can be utilized to identify the position of an object within indoor environments. Over the past years, researchers have proposed various techniques and approaches to tackle this issue and effectively determine or track the location of an object in an indoor environment. These studies can be classified into laboratory experiments (e.g., [18,22]) and real-world cases (e.g., [9,23]), where indoor localization techniques are proposed for controlled and operational environments, respectively. Although laboratory experiments achieve significant localization results, the proposed techniques do not perform as efficiently in real world scenarios. Thus, indoor positioning is a problem that remains unsolved. In the following section, this study briefly presents the most common indoor positioning techniques and provides an overview of the available studies that utilize wireless technologies for localization purposes. We emphasize on works that have applied BLE beacons and the gaps that this work attempts to address is discussed.

### 2.1. Indoor Positioning Techniques

Various techniques have been developed to determine the geometrical placement or position of an object in an indoor environment. The location is estimated by taking into consideration the distance, as well as the angle, between the transmitters and the tracked objects in the environment [24]. In addition, the signal strength is used as a means of creating signatures for each location, in order to identify the location of an object [25,26]. The most common techniques used for indoor localization are triangulation and trilateration [27]. Albeit, techniques such as fingerprinting, proximity [17], dead reckoning [28,29] and other machine learning-based approaches have been developed to mitigate measurement errors. Each technique has certain advantages and disadvantages. Thus, the selection of the most appropriate one highly depends on the application context, while in some cases, using more than one technique and algorithms simultaneously may lead to better performance.

Fingerprinting is the most popular method of localization with high applicability in complex environments due to its high accuracy and low complexity compared with other methods [6,14]. It utilizes the received signal strength (RSS) as an approximate metric to determine the indoor location. To apply the fingerprinting technique, first, the signal strengths are collected by the localization technology (fingerprints) for each possible location of the environment in a database (offline phase) and, thus, the reference fingerprinting map (RFM) [30] is formulated. Then, in the online phase, an object’s signal collected from a location in real time is compared with the fingerprints in the RFM, to solve for the location of the object [18,30]. In this phase, fingerprinting is based on classification algorithms and methods, such as neural networks [11,12], decision trees [23], k-nearest neighbors [31], support vector machines [31] and random forests [32], which predict the object’s current location based on the fingerprint database [33,34]. Since fingerprinting relies on signal strength, the problems that may occur are related to signal variations deriving from communication issues, such as fading, interference or even from environmental factors. 

This research began with tri- and multi-laterations, but the results were very inaccurate. This confirms that they do not fit the BLE beacons due to the noise generated by context factors, hence, signal strength is weaker than expected [35]. Next, fingerprinting along with the classification methods are discussed and, finally, an ensemble method that improves indoor localization in the retail store is proposed.

### 2.2. Wireless Technologies for Indoor Positioning

The technologies used to track an object while it is moving around in indoor environments are mostly (a) Wi-Fi, a popular wireless networking technology [36]; (b) Bluetooth, a wireless technology standard for communication over short distances [37]; (c) RFID, a technology based on radio-frequency identification via electromagnetic fields that can identify and monitor tags that are attached to objects of interest [38]. It is underlined that GPS technology is not appropriate for this purpose, as its signal fails to reach indoor environments [27].

Indoor positioning with Wi-Fi faces the major problem of signal attenuations [39], which is a common cause of faulty measurements. Apart from signal processing works [40], numerous Wi-Fi-based studies have examined indoor positioning approaches [31], reporting either accuracy improvement [8,41,42] or applying machine learning techniques, such as decision trees [23], unsupervised labelling on sequential data [43], unsupervised clustering for multi-floor environments [9] and online sequential extreme learning [44,45]. Bayesian models have also been utilized eliminating the problem of training data. Thus, they are more effective in terms of the cost of gathering data [46]. Other studies have applied fingerprinting [33,47] and classifiers to achieve effective and more accurate Wi-Fi-based indoor positioning [11,48].

Table 1 summarized the Wi-Fi studies with the employed technique.

Radio frequency identification (RFID) technology has also been utilized for indoor positioning and involves curve fitting and location search [18], the hierarchical structure of classifiers [3] and the Kalman-filter drift removal with Heron-bilateration location estimation [49]. In addition, algorithms for higher localization accuracy [5,50,51] have been applied along with deployment innovations [8] (i.e., a spinning RFID antenna).

Table 2 summarized the Wi-Fi studies with the employed technique.

Apart from solely relying on one tracking technology, hybrid implementation approaches have been found in the literature that combine different technologies, such as Wi-Fi and Bluetooth technology [52,53]; Wi-Fi and RGB-D sensors [54] or Wi-Fi, Bluetooth, and long-term evolution (a 4G wireless broadband technology) [55].

Next, the available studies that perform Bluetooth-based indoor positioning are discussed and research gaps that this research addresses are identified.

### 2.3. Blueteooth Technology for Indoor Positioning

Bluetooth is a recent technology in the field of indoor positioning and has been applied both for indoor localization, as well as for location proximity, presenting challenges that should be taken into consideration [56].

More specifically, Bluetooth-based indoor positioning has been conducted by combining BLE beacons with a pedestrian dead reckoning (PDR) technique to provide meter-level positioning and estimate the current position by using a previously determined position [57]. Moreover, they have developed a range-based localization system based on stigmergy that relies on the received signal strength (RSS) of BLE beacon packets [13,58]. On the other hand, Diaz et al. [59] introduced an indoor Bluetooth-based localization system (titled Bluepass) that achieves localization at a room-level based on a signal coverage density method. Similarly, Bobek et al. [60] dealt with the issue of indoor positioning by determining the location at room-level. Further, Liu et al. [16] designed and tested a smart home indoor remote-control system that uses BLE beacons and geomagnetic sensors and proposed a learning indoor location algorithm combining fingerprinting, geomagnetic sensing and PDR to guarantee the accuracy of the location system.

Due to the nature of Bluetooth and the signals emitted, the most widely used and efficient technique used for Bluetooth-based indoor positioning is fingerprinting (e.g., [4,12,13,14,15,61]). For example, Subedi et al. [14] combined fingerprinting with weighted centroid localization aiming to reduce the total number of collected fingerprints to improve the time required for the positioning process. One of the most recent studies combined fingerprinting with geometric techniques to pinpoint the movements of patients in a BLE beacon-enabled hospital room [15].

Moreover, when utilizing Bluetooth signals, a commonly used classification technique for indoor positioning is neural networks. Mazan and Kovarova [12] collected fingerprints (signal strengths by the beacon devices) and employed an artificial neural network to determine the user’s location. Thus, employing multiple neural networks has achieved better accuracy by handling the variability of the strength of the signals transmitted by Bluetooth devices [62].

Table 3 summarized the Bluetooth studies with the employed technique for indoor positioning.

Overall, on the one hand, Wi-Fi technology has dominated the field of indoor positioning until now utilizing a vast variety of techniques. On the other hand, Bluetooth technology has become more mature and reliable for indoor positioning and the available studies are increasing. However, most works are practically laboratory experiments (e.g., [17,18,19,20]) that have not validated their indoor positioning approach in a real, fully operational environment. Further, researchers have experimented with indoor localization of subjects moving only in one-floor contexts (e.g., [56]). Moreover, most studies employ a single positioning technique for performing indoor positioning (e.g., [13,57,63,64]). They do not compare and evaluate different techniques or even combine them to aim for higher efficiency in indoor positioning. On the contrary, they have experimented with a hybrid of different technologies (e.g., Galvan-Tejada et al. [53] proposed an algorithm to obtain the location of a receiver combining Bluetooth and Wi-Fi technologies).

**Figure 1:**
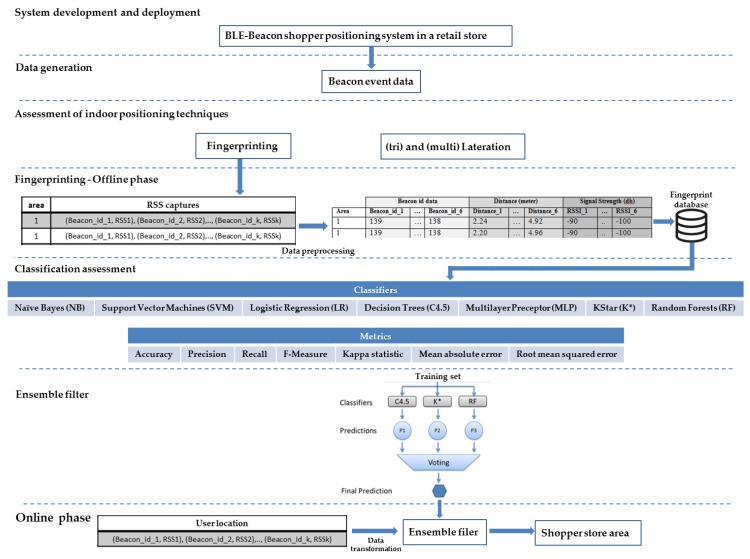
Methodology phases.

To fill these acknowledged gaps in the literature, this study developed and deployed a BLE beacon-based indoor positioning system of shoppers moving in a retail store with two floors of different layout characteristics. The authors experimented with different placement scenarios of the BLE beacons and the ceiling of the store was identified as the most appropriate one. Next, the established localization techniques were applied and evaluated with the purpose of achieving the most efficient position determination of moving customers in the retail store. Tri- and multi-lateration was applied, but the results were disappointing and, then, fingerprinting along with the classification methods were assessed to determine the customer location at the store area-level. The authors did not consider the two floors of the store split into equal smaller areas making a grid (i.e., fixed-length surface) to detect the exact store area of the customer. Instead, the constraints of the store’s physical layout and the retailer’s needs were identified and, thus, a more realistic, variable-length surface model was adopted with store areas of different sizes and dimensions. Then, seven established classifiers were selected and compared with their indoor localization performance in terms of the established metrics of accuracy, precision, recall, F-measure (or F1 score) and Kappa statistic [65,66], as well as mean absolute error and root mean squared error. The variance in parenthesis for each metric was also provided. The random forest algorithm outperformed all the classifiers. A t-paired test was executed employing the F-Measure and the random forest statistically outperformed the other classifiers. In essence, the random forest combines a large number of classifiers targeting better classification results. Thus, it inspired the authors to experiment with an ensemble classification approach targeting the improved localization in the retail store. A hybrid approach was proposed that considers the three best classifiers (random forest, K* and C4.5) based on the above assessment results and executes a voting process taking a weighted vote of their predictions of the customers locations in the store. The absolute mean and root mean squared errors of the ensemble method are significantly lower than the ones of the random forest. Figure 1 summarizes all the above phases of this study.

## 3. A BLE Beacon-Based Indoor Positioning System

In practice, the performance of current indoor positioning techniques was evaluated, as well as the more efficient positioning approaches were pursued through an indoor positioning system that the authors developed. The system developed relies on Bluetooth technology and, specifically, on transmitter devices called beacons that emit radio signals to nearby devices. These signals contain information regarding the identifier of the beacon and other data, such as its RSSI strength. 

**Figure 2:**
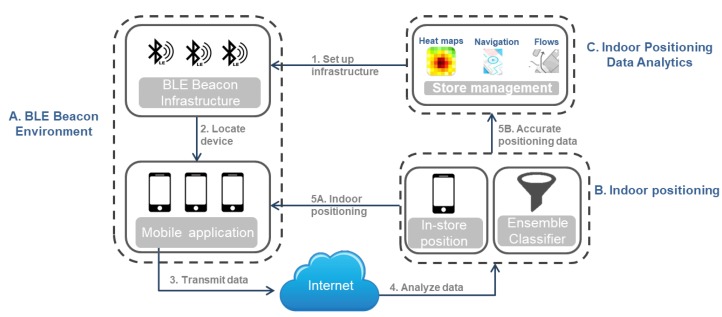
Low-energy Bluetooth (BLE) beacon-based indoor positioning system overview.

Figure 2 depicts the main modules of such indoor positioning systems that utilize BLE beacons: (A) the BLE beacon environment; (B) the indoor positioning module that determines the user’s position based on beacon data captured by the mobile application; (C) the indoor positioning data analytics module that extracts and stores new knowledge from the collected data.

Specifically, the first module (BLE Beacon environment) consists of the following two layers:

(I) The BLE beacon infrastructure layer concerns the beacons that are deployed in the indoor environment (e.g., the beacons’ number and placement) and the data (e.g., beacon device identity) that are transmitted and captured by the mobile applications of the moving users. It is practically the setting upon which the system is deployed. The BLE beacons can be installed in various locations and arrangements depending on the ultimate purpose of the system (e.g., indoor positioning, proximity marketing etc.). Respectively, the BLE beacon manufacturer and the available sensors on the beacon device prescribe the forms of data that the beacons provide, such as the identity (ID) of the beacon device, the strength of the received signal (RSS), the distance between the beacon and the mobile device of the user and more information, such as light strength and humidity or temperature.

(II) The mobile application layer refers to an application that captures the beacon data during the indoor movement of the users. It transmits them via the internet to the back-end cloud infrastructure, which stores the received data from the BLE beacons and the identity of the mobile application. Then, the mobile application receives the user’s position in the indoor environment from the back-end infrastructure each time that a request is sent with the captured information from the BLE beacons.

Specifically, each user’s visit in the indoor environment under study is recorded as a single user session, which contains all the data gathered from the user’s mobile device while the user moves indoors and passes through the installed beacons. The mobile application is programmed to capture transmissions from nearby beacons in order to determine the user’s position. These snapshots are called events and are contained in each session. Each captured event by the application contains (i) the beacon ID (the unique identifier of the beacon), (ii) the signal strength (RSSI) that the application captures from the specific beacon, in order to determine how close or how far the mobile device from the transmitter is, and (iii) the distance between the beacon and the moving device. The application can capture events from more than 6 beacons, which are then stored in the back-end’s database. The data from the closest beacons to the mobile device are utilized to determine the user’s location.

Next, the indoor positioning module, implemented in the backend, first collects and stores the pre-mentioned beacon data generated during the user’s sessions and the identity of the user’s mobile device. Then, the key component of this module, i.e., the indoor positioning technique, processes and filters the beacon events’ data and determines the indoor position of the user’s device, namely the user’s position.

Finally, the purpose of the indoor positioning data analytics module is to further analyze the recorded data that reflects the users’ movements in the indoor environment as captured by the beacon devices in collaboration with the mobile application. This module generates new insights and knowledge describing the users’ indoor movements e.g., the users’ traffic heat maps and the users’ navigation flows. The outcomes of this module can be exploited by other applications, such as a security system of a building that needs to know the visitors traffic patterns.

This research concerns the indoor positioning module and, specifically, the positioning technique it executes. This study evaluates the available techniques and proposes ones that perform efficient indoor positioning. To this end, a real BLE Beacon-based indoor positioning system in a grocery retail store that monitors shoppers holding mobile devices was designed and deployed. The generated beacon data was utilized to assess indoor positioning techniques and propose a hybrid approach that improves indoor localization of consumers in the store.

## 4. Case Study: A BLE Beacon-Based Shopper Positioning System in a Grocery Retail Store

A BLE beacon-based indoor positioning system was deployed, such as the one described above, in a grocery retail store. A retail store was selected as a test bed for evaluating the indoor positioning techniques because a retail store is a challenging environment due to many factors that could affect the localization accuracy, such as obstacles in the stores (e.g., walls or product signs), product materials (e.g., liquids) that interact with the emitted signals or even the consumers moving in the store. The more challenging the environment, the more generalizable and valid the evaluation results of the indoor positioning techniques and the performance of the proposed positioning approach. Specifically, the store application area consists of two floors (ground floor and first floor) with different layout characteristics. This study installed 81 BLE Estimote beacon transmitters. The utilized Bluetooth version was 4.2 LE standard, as it was supported by the deployed beacons. The more recent protocol (i.e., Bluetooth 5) was not used as it was not supported by the beacon transmitters available.

Considering the mobile application layer of the shopper positioning system, a mobile application was developed for shoppers to download in their Android smart phone devices. It interacts with the beacons while the shopper moves in the store. During each shopper visit (i.e., a shopper session), the mobile device captures all the data (events) from the beacons installed in the area into which the shopper moves. Each event consists of the beacon ID, the beacon’s signal strength RSSI and the distance between the beacon and the mobile phone. Several experiments were performed using event data from different numbers of beacons to estimate the consumer’s in-store location. Utilizing the six closest beacons to the consumer’s smart phone proved to be the most effective manner to identify the shopper’s position. This is also aligned with the best practices described in Estimote’s forums (https://forums.estimote.com).

Next, the system’s BLE beacon infrastructure layer is described, namely where the available BLE beacons were placed in the store area. This is emphasized because it affects the accuracy of the captured shopper data and, consequently, the performance of the indoor positioning technique that determines the shopper’s indoor location

### Map and BLE Beacons Placement

The system was deployed in a store area of two floors of 1200 m^2^ each. On the one hand, these two floors were selected due to the business purpose of the retailer, which concerned consumer traffic heatmaps showing how the consumers move within these two floors of the grocery store. Next, the ground floor was divided into 23 areas and the first floor in 30 areas, based on the business purpose and the technical efficiency. 

The literature proposes several approaches to determine an appropriate transmitter placement within an indoor environment [67,68]. Drawing on them, this study aimed to place the available 81 beacon transmitters in such a manner that virtual perimeters were created around the consumers per store area, in order for the location of the consumer to be determined more efficiently. To this end, different placement scenarios of the beacons were experimented with in order to detect the most appropriate one. 

**Figure 3:**
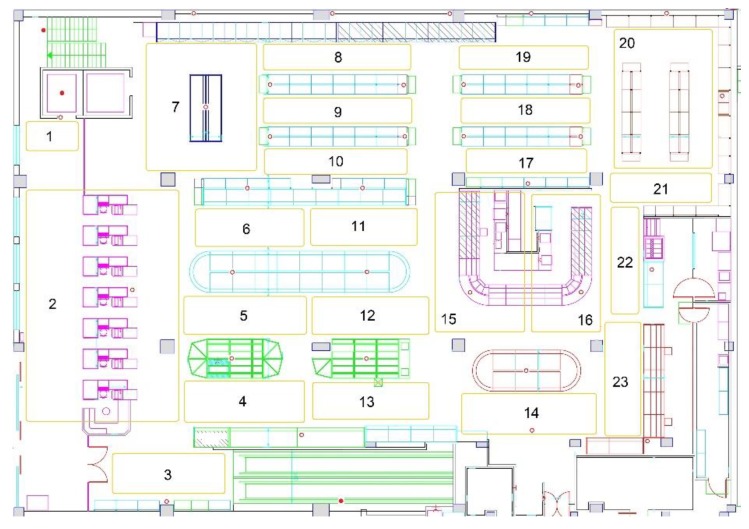
Map of the BLE beacon-enabled ground floor of the grocery store (store areas are labelled with a number from 1 to 23, beacons are presented in red circles, elevator beacons are filled with red color).

**Figure 4:**
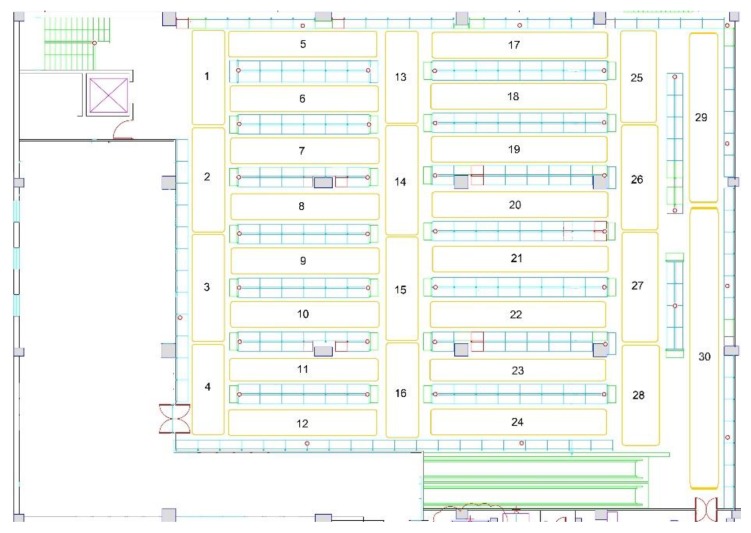
Map of the BLE beacon-enabled first floor of the grocery store (store areas are labelled with a number from 1 to 30, beacons are presented in red circles).

On the one hand, various placements of the transmitters on the top of the store aisles were tested. However, various obstacles (e.g., products that clerks placed over the transmitters during the shelf replenishment process), which covered the beacons, prevented capturing the beacons’ signals. On the other hand, alternative scenarios were experimented with by putting the transmitters on the ceiling. Overall, the placement on the ceiling was more successful, as it was unlikely for beacons to be covered by products, thus preventing their signal from being efficiently emitted. Figure 3 and Figure 4 present the final beacons placement in the ground floor’s and the first floor’s ceiling, respectively. Each area in a floor is indicated with a number (1–23 for ground floor, 1–30 for first floor) and each transmitter is denoted with a red circle. The red circles filled with red color are the beacons in the elevator, which are used to track the consumers’ transition between the two floors. This final deployment facilitates the visibility of the moving shoppers from all the surrounding transmitters and ensures that more accurate shopper event data are received by the indoor positioning module for determining the shoppers’ location.

The next section presents the evaluation study of indoor positioning techniques using the beacon data reflecting the shoppers’ movements in the retail store, as well as the proposed hybrid approach.

## 5. Assessment of Indoor Positioning Techniques

This study tested and evaluated the most common and established localization techniques referred to in Section 2.1, Indoor Positioning Techniques. At the beginning, tri- and multi-laterations were applied, but they did not achieve satisfying results. Next, this study experimented with fingerprinting. The following subsections present the evaluation results which motivated the proposed approach that achieves more efficient position determination.

### 5.1. Trilateration

To perform trilateration for specifying the exact location of the consumer in the store, the exact coordinates of all beacons/transmitters were marked to calculate the consumer’s position. Subsequently, based on the distance between the beacon and the shopper’s mobile phone indicated by each event, the coordinates of the moving consumer were calculated.

More specifically, to calculate the position of the shopper, the following steps were performed: (i) Bluetooth synchronization; (ii) time calculation; (iii) noise elimination. Regarding Bluetooth synchronization, BLE beacons are to set to emit signals every 0.5 s. The emitted signal contains information regarding the ID of the BLE beacon and when it is received by shopper’s mobile application. The captured data include the ID, the received signal strength and the distance between the mobile device and the BLE Beacon. 

The mobile application synchronizes the received Bluetooth signals and records the top 10 BLE beacons with the strongest signal strength that are closest to the shopper’s device. In order to avoid outdated data, in the time calculation, the mobile application discards data that are older than the rest of the captured.

This study began with event data from the three closest beacons in distance and more beacons were experimented with until reaching six beacons (i.e., (multi)lateration) to calculate the shopper’s coordinates. 

The beacon transmitters used provided, via their software development kit (SDK) (https://developer.estimote.com), the distance between the shopper’s device and the beacon. As a result, trilateration was performed based on the respective distances. 

**Figure 5:**
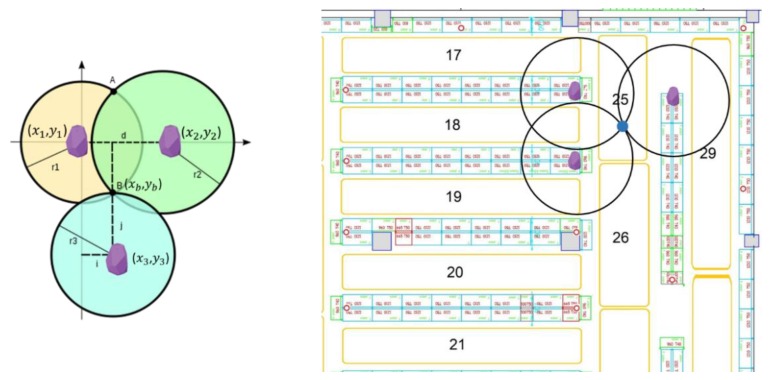
Shopper position calculation using trilateration.

As shown in Figure 5, the coordinates of each beacon (i.e., $Beacon_{1}$($x_{1},\;y_{1}$), $Beacon_{2}$($x_{2},\;y_{2}$), $Beacon_{3}$($x_{3},\;y_{3}$)) were utilized to determine the coordinates of the target point B, which indicates the location of the shopper. In order to calculate the coordinates of B($x_{b}$), it is treated as the intersection of 3 circles, whose centers are the locations of the 3 beacons $Beacon_{i}$($x_{i},\;y_{i}$), for i = 1, 2, 3. The distances between each beacon and the shopper are the radii of each circle. For each circle the equation is:

$${\left(x-x_{i}\right)}^{2}+{\left(y-y_{i}\right)}^{2}=r_{i}{}^{2}\;.\;$$

By expanding out the squares for each one of the three equations:

(1) $$x^{2}-2x_{1}x+\;x_{1}{}^{2}+\;y^{2}-2y_{1}y\;+\;y_{1}{}^{2}=\;r_{1}{}^{2}$$

(2) $$x^{2}-2x_{2}x+\;x_{2}{}^{2}+\;y^{2}-2y_{2}y\;+\;y_{2}{}^{2}=\;r_{2}{}^{2}$$

(3) $$x^{2}-2x_{3}x+\;x_{3}{}^{2}+\;y^{2}-2y_{3}y\;+\;y_{3}{}^{2}=\;r_{3}{}^{2}$$

Subtracting Equation (2) from Equation (1):

(4) $$(-2x_{1}+\;2x_{2})x\;+\;(-2y_{1}+\;2y_{2})y\;=\;r_{1}{}^{2}-\;r_{2}{}^{2}-\;x_{1}{}^{2}+\;x_{2}{}^{2}-y_{1}{}^{2}+y_{2}{}^{2}$$

Next, subtracting Equation (3) from Equation (2):

(5) $$(-2x_{2}+\;2x_{3})x\;+\;(-2y_{2}+\;2y_{3})y\;=\;r_{2}{}^{2}-\;r_{3}{}^{2}-\;x_{2}{}^{2}+\;x_{3}{}^{2}-y_{2}{}^{2}+y_{3}{}^{2}$$

Equations (4) and (5) form a system of two equations with two unknowns, x and y:

Ax + By = C

Dx + Ey = F

The solution of the system is:

$$x\;=\frac{CE-FD}{EA-BD}\;\mathrm{and}\;y\;=\frac{CD-AF}{BD-AE}$$

Respectively for multilateration, a similar approach was followed for 6 beacons and, then, a linear least squares solver was applied to get the (x,y) solution of the equations system. 

However, the results were very inaccurate. Trilateration and multilateration can hardly be used with BLE beacons due to the noise generated by factors, such as product interference. Hence, the signal strength, which can be easily affected, is weaker than expected [35]. The distance from a BLE beacon is calculated based on a function that receives the signal strength as an input and provides the estimated distance as the output. Thus, in most cases, the distance deviates from the expected and it becomes impossible to have an accurate estimation. 

**Figure 6:**
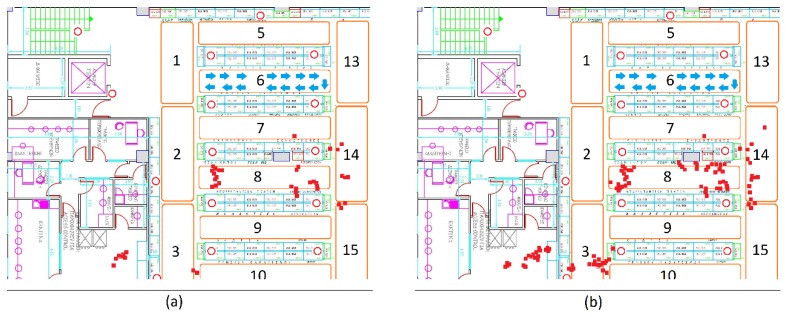
Tri- and multi-lateration performance in the retail store with (**a**) 3 and (**b**) 6 beacons.

Figure 6 presents two indicative evaluation cases of trilateration and multilateration in the store, respectively. The blue arrow in both examples (a and b) highlights a shopper’s route that he moved across aisle 6 and, then, returned to the point where the shopper initially started. In the first case (Figure 6a), the data from the closest (in distance) to the shopper’s mobile device three beacons was utilized. Notably, the red dots, which are the outcome of the trilateration function, fall into an entirely different aisle and even outside the boundaries of the floor (bottom-left red dots). The distance error between the actual consumer’s position and the estimated varies from 5 to 10 m. In the second case (Figure 6), the six closest beacons were used to identify the shopper’s location. Similarly, this study found the consumer to be in entirely different areas than the actual ones (see the red dots). To improve the results, additional logic was even employed for manipulating the trilateration data, such as rejecting results (dots in the figures) that are not valid or have major deviations from the previous estimations. Nonetheless, the results were not significantly improved, as they were already very inaccurate. Thus, the authors realized that both trilateration and multilateration are not appropriate approaches for accurate indoor positioning in a retail store.

### 5.2. Fingerprinting

Then, the fingerprinting approach was applied by calculating the distance between the BLE beacon and the tracked consumer, not on the x-y coordinates-level, but on area-level. To be more specific, the beacons’ signal strength and the nearby beacon IDs were utilized to determine the location of a consumer at the area-level, namely in the aforementioned store areas that were defined with the beacons’ placements.

To formulate the reference fingerprinting map (RFM), two vectors were utilized that contain the store areas and the BLE beacons. Vector A = {$area_{i}$ | i = 1,…,53} was used that contains the 53 store areas were defined for both store floors and vector B = {$beacon_{j}\left(x_{j},\;y_{j}\right)$ | j = 1,…,81} that reflects the exact locations of the deployed BLE beacons. For each of the areas, the RSS fingerprints were recorded at time instances $t_{m}$, with m = 1,…,M and were organized in the following fingerprinting map, that contains the RSS values for all store areas from all BLE beacons at the time instant $t_{m}$.

$$RFM\left(t_{m}\right)\;=\;=\;\left(\begin{array}{cccc} RSS_{1}^{1}\left(t_{m}\right) & RSS_{2}^{1}\left(t_{m}\right) & \cdots & RSS_{j}^{1}\left(t_{m}\right)\\ RSS_{1}^{2}\left(t_{m}\right) & RSS_{2}^{2}\left(t_{m}\right) & \cdots & RSS_{j}^{2}\left(t_{m}\right)\\ \vdots & \vdots & \vdots & \vdots \\ RSS_{1}^{i}\left(t_{m}\right) & RSS_{2}^{i}\left(t_{m}\right) & \cdots & RSS_{j}^{i}\left(t_{m}\right) \end{array}\right)\;,\;m\;=\;1,..,M\;,\;i\;=\;1,..,23\;,\;j\;=\;1,..,81$$

The reference fingerprinting map RFM is a sparse array, as each area is not covered by all the available BLE beacons. In addition, RFM contains the parameter of time. In addition, if the captures of different timestamps are compared, how the shopper moved can be identified, based on the captured RSS values. The matrix RFM was utilized for collecting the dataset of the offline fingerprinting phase.

During the offline phase of fingerprinting, a dataset was collected that reflected the beacons’ signal strength in the store areas, as well as the distance of the tracked object (consumer’s mobile) from a transmitter/beacon. Two researchers were moving around in the two floors of the store for two hours holding two mobile devices. The first device was a Samsung Galaxy J5 and the second one was a Samsung A3 and both devices were using Android version 6.0.1. Further, 7.000 events generated from the closest six beacons (in distance) to the researchers’ mobile device were gathered during their simultaneous movements in the two floors of the store. The collected data was stored in a table, where every row was a single event relating the store area where the researcher was with the installed beacons (beacon IDs), the distance from each beacon/transmitter and the signal strength (see Table 4).

Then, the collected events were utilized as training data for training the classification algorithms (classifiers) in predicting the store area where the consumer was located. The output of the algorithms is the store area and the input are the afore-described collected data (beacon IDs, distance and signal strength per store area) reflecting movements in the store.

Table 4 exhibits the structure of the final data input of the different classification algorithms. The store area is the dependent variable (output of the algorithm) and has 53 alternative values (class labels) that correspond to the areas/regions which divided the two store floors (as Figure 3 and Figure 4 depicts). To ensure high internal validity of the experimentation, an efficient sampling process was needed. Thus, sufficient events were captured for all store areas based on their surface in m^2^. The larger areas were represented by more events as seen in Table 5. The area size varies from relatively large (57 m^2^) to small (9 m^2^) with the first floor having a grid layout, while the ground floor is a mix of grid and open layouts. Next, the classification algorithms used are presented and their classification capabilities are compared.

### 5.3. Fingerprinting: Classifiers’ Assessment and Comparison

The most commonly used classifiers were selected, which have already been mentioned. Indeed, the following seven approaches were executed: (i) Naïve Bayes (NB) [69]; (ii) support vector machines (SVM) [70]; (iii) logistic regression (LR) [71]; (iv) decision trees (C4.5) [72]; (v) multilayer perceptron neural networks model (MLP) [73]; (vi) KStar (K*) [74] and (vii) random forests (RF) [32]. Naïve Bayes was selected as it is one of the simplest classification algorithms with strong independence assumptions between the features. Additionally, logistic regression was selected to examine the classification problem as a generalized linear model and KStar, an entropy-based algorithm for investigating their performance in such classification cases. Weka software was used for our experiments [75]. 

Specifically, this study assessed and compared the performance of the classifiers based on the established metrics of accuracy, precision, recall and f-measure (or F1 score) [65]. Accuracy measures the number of correct classifications performed by the classifier.

$$\mathrm{Accuracy}\;=\;\frac{True\;Positive+True\;Negative}{True\;Positive+True\;Negative+False\;Positive+False\;Negative}$$

Precision indicates the exactness of the classifier, meaning that higher and lower precision leads to less and more false positive classifications, respectively.

$$\mathrm{Precision}\;=\;\frac{True\;Positive}{True\;Positive+False\;Positive}$$

Recall measures the classifier’s completeness. Higher and lower recall means less and more false negative classifications (the captured events are not assigned as related to a store area, although they should be), respectively.

$$\mathrm{Recall}\;=\;\frac{True\;Positive}{True\;Positive+False\;Negative}$$

Precision and recall are increased at the expense of each other. Thus, they were combined to produce the weighted harmonic mean of both metrics, which is the F-measure.

$$F-\mathrm{Measure}\;=\;2*\frac{Recall*Precision}{Recall+Precision}$$

Further, the Kappa statistic metric was utilized. This measures how closely the captured events classified by the algorithm match the true events, controlling for the accuracy of a random classifier as measured by the expected accuracy [66]. In other words, it shows how much better the assessed classifier is performing over a classifier that simply guesses at random according to the frequency of each class.

The evaluation was performed via a 10-fold cross-validation [76], where the original aforementioned collected events/data were randomly divided into ten equal subsets. Of these ten sub-sets, one is retained as the validation test of the classifier and the remaining nine ones are used as training data. Table 6 includes the assessment results in terms of the above metrics and mean absolute error and root mean squared error. The values in Table 6 are the weighted averages of all classes (i.e., the store areas) in order to acquire a more compact interpretation of the performance of each classifier. The variance in parenthesis for each metric was also provided to depict how it fluctuates. The results show that the random forest algorithm outperforms all the other algorithms in most of the metrics.

Moreover, a t-paired test was utilized to explore which classifiers were significantly better than the others (a = 0.05 level) [77]. The F-Measure was employed to compare them because it is more reliable and provides a good trade-off between precision and recall. Again, random forest statistically outperforms the other classifiers, followed by K*, C4.5, multilayer perceptron, logistic regression, Naïve Bayes and support vector machines in descending order. Random forest is by design an ensemble learning method, meaning that it combines a large number of classifiers targeting better classification results. Therefore, the performance of random forest inspired the authors to explore the potential of an ensemble classification approach that follows the design principles of random forest.

### 5.4. Proposed Hybrid Approach: An Ensemble Filter

**Figure 7:**
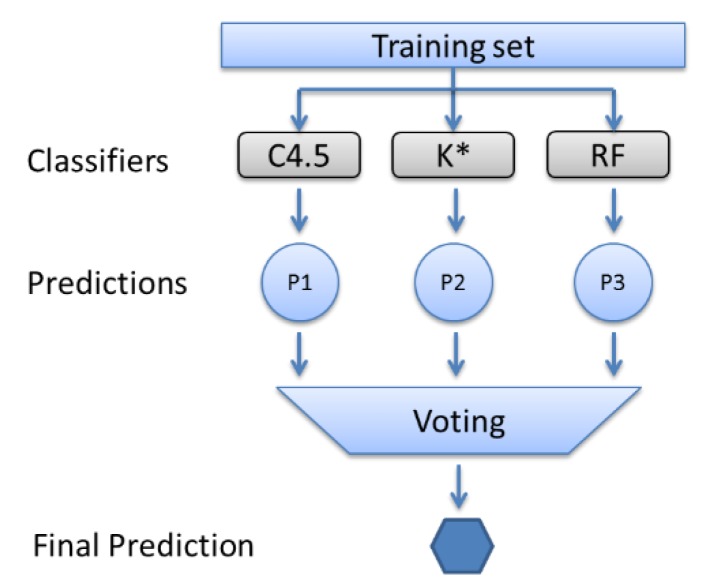
The Proposed ensemble classifier.

Instead of relying solely on the most efficient classifier for our case (as shown, that is the random forest), a hybrid approach was proposed that executes a voting process involving a set of multiple classifiers. In essence, this study suggests an ensemble method [78,79] that runs the three significantly better classifiers based on their afore-assessed F-measure metric, assessed in the previous step (Section 5.3), and takes a weighted vote of their predictions (see Figure 7).

More specifically, the proposed ensemble filter is a meta-classifier that performs weighted majority voting among the three selected classifiers (i.e., C4.5, K* and random forest). The ensemble filter predicts the class label y. To do so, a weight $w_{j}$ is associated with each classifier $C_{j}$. The filter formula is the following:

$$y\;=\;argmax_{i}\sum_{j=1}^{m}w_{j}{Χ}_{A}(C_{j}\left(x\right)=i),$$

where ${Χ}_{A}$ is the classification function $\left[C_{j}\left(x\right)=i\;\in A\right]$ and A is the set of unique class labels (i.e., the store areas). The outcome of the formula is the class with the arguments with the greater weight (i.e., argmax). In this case, the weights are assigned automatically via the Weka software.

Similar to the previous step, the three more efficient classifiers were utilized and a new classifier was devised. The evaluation is performed as before via a 10-fold cross-validation [76] and the performance of the suggested ensemble filter is presented in Table 7 compared to the three classifiers used. 

To assess the indoor positioning capability of our ensemble method, this study compared the actual locations of the consumers in the store that the aforementioned collected data show with their final predicted locations (estimations of store areas) by the proposed ensemble. Each classifier predicts its output and then weighted majority voting is performed to predict the area of the shopper.

Table 7 shows that the ensemble method achieves a slightly lower accuracy than the best classifier (i.e., random forest). Most importantly, the absolute mean and root mean squared errors are significantly lower (40.7% and 18% lower, respectively). Lower errors mean that the result is closer to the actual one, thus leading to more efficient location determination.

**Figure 8:**
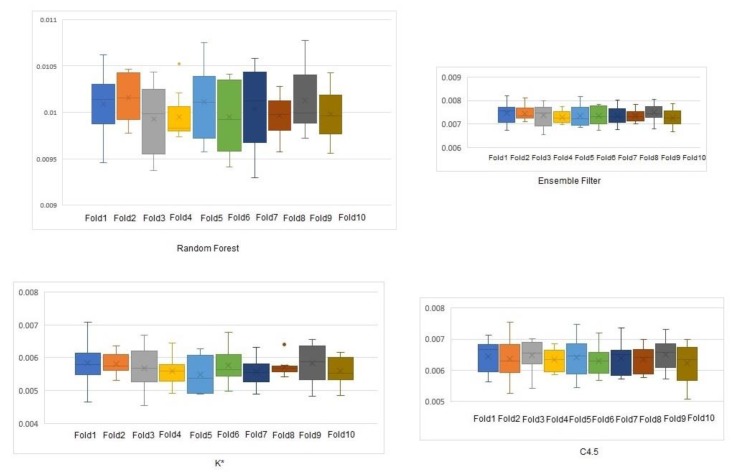
Absolute mean error of the ensemble method and the three classifiers.

**Figure 9:**
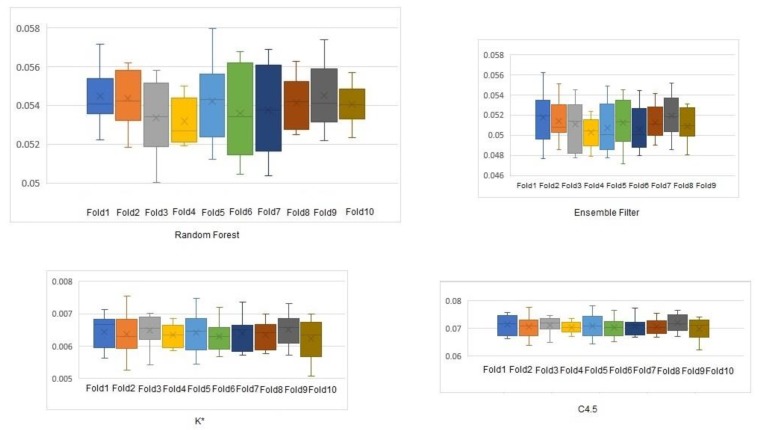
Root mean squared error of the ensemble method and the three classifiers.

The absolute mean error (Figure 8) and root mean square error (Figure 9) of the three classifiers and our filter were plotted to show their variations. Boxplots were used to depict the error variance at each step of the 10-fold cross validation. 

**Figure 10:**
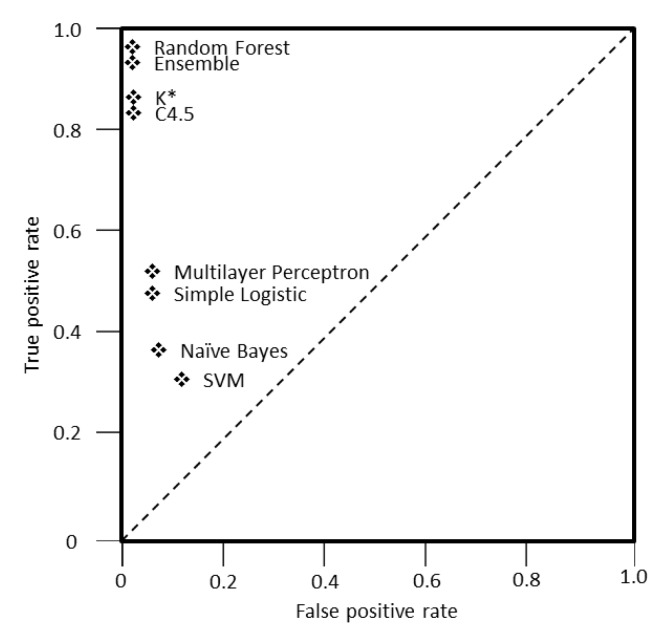
Classifiers receiver operating characteristic (ROC) space graph.

For a better visualization of the performance of all classifiers examined, this study decided to utilize receiver operating characteristic (ROC) graphs, which is a technique usually adopted in machine learning and data mining [80]. Figure 10 presents the trade-off between the true positive and false positive rate for each classifier. The important points in this graph are (a) point (0,0), which means that the classifier commits no false positive errors, but also gains no true positives, (b) point (1,1) where the classifier commits positive classifications unconditionally and (c) point (0,1), representing a perfect classification [80]. A classifier is considered to perform better the more it moves to the north-west part of the graph and also makes positive classifications only with strong evidence, as the false positive rate gets close to the Y axis [80].

As shown, the random forest classifier and the ensemble approach are close to the upper left point of the graph, namely they make positive predictions based on strong evidence, followed by K* and C4.5. The rest of the assessed classifiers (i.e., multilayer perceptron, simple logistic, Naïve Bayes and support vector machines) tend to have a lower performance than the first ones as their distance from point (0,1) increases.

**Figure 11:**
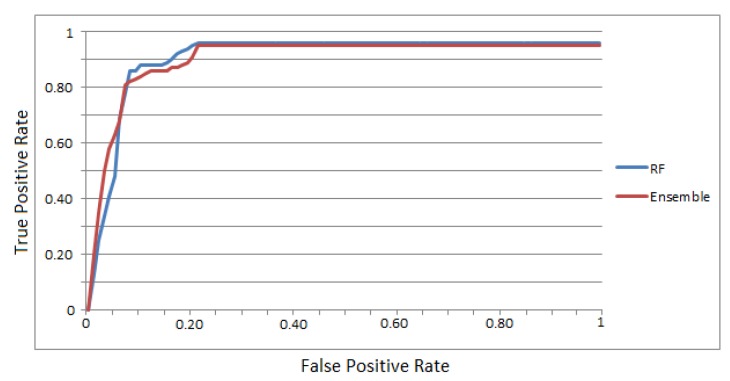
ROC curve of random forest and the proposed ensemble method.

For further comparison between the two best methods (random forest and our ensemble classifier), the multiclass classification of the consumer’s area was transformed into a binary classification to evaluate the capability of only these two classifiers to correctly predict the consumer’s position. Figure 11 presents the ROC curve of the random forest and ensemble classifiers and demonstrates their training speed. The ensemble classifier is characterized by a better learning ability as the training instances increase up to 0.65 of the true positive rate. From 0.65 to 0.80, both algorithms tend to behave the same and above 0.8, the random forest is slightly better. When both algorithms reach 0.95 of the true positive rate, they have a similar behavior, with the random forest being slightly superior by 0.01.

**Figure 12:**
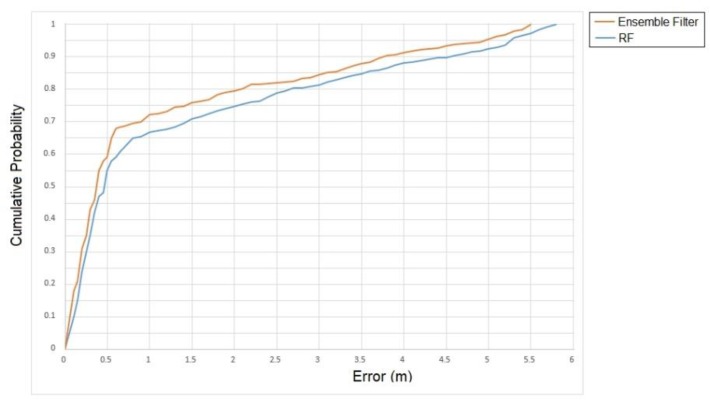
Positioning error for the proposed ensemble filter.

Finally, Figure 12 presents the overall cumulative probability and error correlation of the proposed method and the random forest. Based on our experimentation, it was concluded that for approximately 70% of the cases (captured events of consumers), the ensemble method results in a localization less than 1 m and in 80% of the cases, the localization error is approximately 2 m. The results confirm prior similar studies that try to improve the detection accuracy when using BLE beacons. On the contrary, for the random forest classifier, in 80% of the cases the localization error is approximately 2.5 m. In retail environments in particular, such deviation is significant, because even 0.5 m away from the actual shopper’s position may lead to position him in a different shopping isle and in front of a different store shelve, thus a different product category. Considering the need of retailers to use such indoor positioning systems for knowing the actual shopping trips of customers and offering them personalized services (e.g., promotions designed based on their route in the store), the lower positioning error of our ensemble filter compared to the random forest is significant even though they do not have significant differences in terms of accuracy and F-measure.

## 6. Discussion

Most indoor localization studies are laboratory experiments (e.g., [5,19,20]). For example, Subhan et al. [13] achieved a localization error to 2.67 m [13], while broader experimental environments achieved 2.6 m [81]. However, high performance is not guaranteed in the respective real, fully operational environment. However, this study’s approach concerns a real-world case with much more BLE beacon transmitters and achieves better localization that varies from 2 to 2.5 m. These results are encouraging considering such environments as retail stores impose several restrictions to experimentation. For example, there are various sources of noise that affect the signal quality and, as a result, the efficiency of indoor positioning (e.g., the moving shoppers and the density of shoppers in the store affect the emitted signals). In addition, the mobile devices of the shoppers have different kind of wireless cards and interpret signal strength differently. This may lead to misreading the signal strength and, thus, to inaccurate data. Finally, the store layout and the contained products can affect signal quality. 

To the best of the authors’ knowledge, this is the first work concerning indoor localization of consumers in a fully operational retail store with two floors. Yohan et al. [64] applied BLE technology and proposed a secure indoor positioning-based mobile system for a retail store. They contributed with an indoor positioning-based mobile payment authentication protocol and evaluated its security strength. The proposed system applies two well-known indoor positioning algorithms (least squares and trilateration) and they do not work on the improvement of indoor positioning of the customers. On the contrary, this study focused on the achievement of the best possible indoor localization of consumers. To this end, different indoor positioning techniques were evaluated and compared and, finally, a hybrid approach was proposed that offers better indoor localization results.

Further, for indoor positioning, researchers usually split the initial surface into equal smaller areas (fixed-length surface), thus forming a grid which can then be used to detect the exact area where the object is located. However, as Wang et al. [18] suggested an important limitation of fingerprinting which relies on the assumption that indoor (sub) areas are regularly formed (e.g., constitute a perfect square). Thus, this study took under consideration the technology limitations of BLE beacons, as well as the challenging physical layout of a retail store with different sized areas, and selected store areas of different sizes and dimensions (variable-length surface). The special business needs of the retailer guided the final segmentation of the areas and their dimensions. For example, the retailer is more interested to know whether the consumers pass often and stay in store areas with popular, fast-selling products.

Regarding the practical implications of this study, the proposed indoor positioning system can be the cornerstone of various location-based services for consumers in retail stores. For example, the ability of one-to-one customer tracking and identification, which was not available with technologies such as Wi-Fi and RFID, can enable personalization and, thus, improved shopper experience by providing personalized offers, coupons, or even content. Moreover, the analysis of the recorded consumers’ movements in the store can provide new, valuable insights on the consumers’ behavior. For example, heatmaps depicting the areas that the shoppers spend the most time, and/or the areas that the most shoppers pass by can be very helpful for marketing and store managers for designing effective marketing actions and in-store advertisements, or even identifying selling gaps by monitoring the areas that shoppers spend considerable time, but finally do not purchase the products displayed in them. 

## 7. Conclusions

This paper addresses customers’ indoor positioning in a real grocery retail store with two floors via a BLE beacon-based indoor positioning system. Targeting to achieve the most efficient position determination of the moving customers, fingerprinting was first applied and, then, the indoor localization performance of seven established classifiers was compared to determine the customer location at the store area-level. The random forest algorithm outperformed all the classifiers and inspired the authors to build an ensemble classification approach that can achieve even better localization in the retail store. The absolute mean and root mean squared errors of this ensemble method are significantly lower than the ones of the random forest (40.7% and 18% better, respectively).

This study is not without limitations. For example, the authors started by assessing the performance of the most common techniques for indoor positioning (i.e., trilateration and fingerprinting). Fingerprinting may be a widely used technique, however it faces several limitations when applied on dynamically changing environments [82,83], such as retail stores. One of the major disadvantages of fingerprinting is the maintenance and updating of the reference fingerprinting map, which is a labor intensive and time-consuming task. Further disadvantages are the number of access points required to perform efficient fingerprinting (in order to cover the store surface) and the effects of mobile devices on the received signal strength, as the signal measurement at the same location by different devices may vary significantly.

Inspired by the fingerprinting disadvantages, future research may concern the assessment of more advanced approaches (e.g., [30,61]) that aim to eliminate noise and provide more efficient results. The authors also plan to test a hybrid approach that relies both on BLE beacons and Wi-Fi technology with the purpose of exploring the impact on the indoor localization efficiency.

## Figures and Tables

**Table 1:** Overview of studies that utilize Wi-Fi technology for indoor positioning.

Authors	Techniques
Lin and Lin (2005)	Fingerprinting
Liu et al. (2007)	Triangulation /Fingerprinting /Proximity
Yim J. (2008)	Decision trees
Campos et al. (2014)	Fingerprinting
Galván-Tejada et al. (2014)	Random Forest /KNN/Neural Networks
Morales et al. (2014)	Faulty measurements
Zou et al. (2014;2015)	Online Sequential Extreme Learning
Torres-Sospedra et al. (2015)	Fingerprinting
Perez et al. (2016)	Unsupervised learning
Turgut et al. (2016)	Trilateration/Fingerprinting /Proximity/PDR/Hybrid
Duque Domingo et al. (2017)	Fingerprinting, Synchronized Euclidean distance
Cao et al. (2019)	Fingerprinting
Wang et al. (2019)	Fingerprinting/Clustering/signal weighted Euclidean distance
Zhang et al. (2019)	Convolutional Neural Network/Gaussian Process Regression

**Table 2:** Overview of studies that utilize radio frequency identification (RFID) technology for indoor positioning.

Authors	Techniques
Zou et al. (2013)	Extreme Learning
Keller et al. (2014)	Classification
Montaser & Moselhi (2014)	Triangulation/Proximity
Calderoni et al. (2015)	Random Forest classifiers
Huang et al. (2015)	Kalman-filter drift removal/Heron-bilateration
Wang et al. (2015)	Curve fitting
Xu et al. (2017)	Bayesian probability and K-Nearest Neighbor
Liu, F. and Zhong, D. (2018)	Glowworm Swarm Optimization with semi-supervised online sequential extreme learning
Shen et al. (2019)	Angle of arrival method and spinning antenna

**Table 3:** Overview of studies that utilize Bluetooth technology for indoor positioning.

Authors	Techniques
Altini et al. (2010)	Neural Networks
Diaz et al. (2010)	Signal Coverage Density Method
Subhan et al. (2011)	Fingerprinting
Cabero et al. (2014)	Proximity
Kim et al. (2015)	Time windows and frequency
Li et al. (2015)	Dead Reckoning
Palumbo et al. (2015)	Stigmergy
Mazan and Kovarova (2015)	Neural Networks
Bobek et al. (2015)	Rule learning
Faragher and Harle (2015)	Fingerprinting
Paek et al. (2016)	Geometric Adjustment
Kriz et al. (2016)	Fingerprinting
Castillo-Cara et al. (2017)	Supervised learning
Liu et al. (2018)	Fingerprinting, Dead Reckoning
Sung et al. (2018)	Fingerprinting, Dead Reckoning, Kalman filter
Yohan et al. (2018)	Trilateration, Least squares
Ferreira et al. (2018)	Geometric Adjustment
Zuo et al. (2018)	Graph optimization
Ke et al. (2018)	Multilateration
Subedi & Pyun (2019)	Fingerprinting
AL-Madani et al. (2019)	Fuzzy logic
Mohsin et al. (2019)	Fingerprinting

**Table 4:** Structure of the collected dataset in the fingerprinting offline phase.

	Beacon id data			Distance (meter)			Signal Strength (db)		
Area	Beacon_id_1	…	Beacon_id_6	Distance_1	…	Distance_6	RSSI_1	…	RSSI_6
12	139	…	138	2.24	…	4.92	−90	…	−100
24	164	…	203	1.06	…	4.87	−81	…	−101
…	…	…	…	…	…	…	…	…	…
5	152	…	149	1.67	…	4.85	−86	…	−99

**Table 5:** Fingerprinting data sampling.

Area Size (m^2^)	Number of Areas	Store Areas	Floor	Captured Events
57.5	1	20	Ground	154
50	1	7	Ground	150
44	1	2	Ground	165
33	2	15,16	Ground	143
28	1	30	First	162
19.5	1	29	First	148
19	1	23	Ground	80
16	16	5-12,17-24	First	146
15.5	6	8-10,17-19	Ground	115
12.5	8	3-6,11-14	Ground	123
9.5	12	1-4,13-16,25-28	First	75
5	2	21,22	Ground	71

**Table 6:** Assessment results of classifiers.

	Classification Algorithms (Classifiers)						
Assessment Metrics	NB	SVM	LR	C4.5	MLP	K*	RF
Accuracy	74.08% (1.73)	85.11% (1.48)	85.92% (1.70)	86.84% (1.36)	90.87% (2.01)	93.51% (1.33)	95.95% (1.06)
F-Measure	0.744 (0.10)	0.850 (0.08)	0.859 (0.09)	0.868 (0.06)	0.908 (0.13)	0.935 (0.05)	0.959 (0.03)
Kappa statistic	0.7341 (0.02)	0.8477 (0.01)	0.8564 (0.02)	0.8651 (0.01)	0.9062 (0.01)	0.9336 (0.01)	0.9586 (0.01)
Mean Absolute Error	0.0101 (0.03)	0.0371 (0.02)	0.0056 (0.02)	0.0056 (0.01)	0.0043 (0.02)	0.0026 (0.01)	0.0086 (0.01)
Root Mean Squared Error	0.08891 (0.03)	0.1351 (0.02)	0.0592 (0.02)	0.0662 (0.01)	0.0543 (0.02)	0.0439 (0.01)	0.0478 (0.01)

**Table 7:** Assessment results of the proposed ensemble method.

Classifiers				
Assessment Metrics	C4.5	K*	RF	Ensemble
**Accuracy**	86.84% (1.36)	93.51% (1.33)	95.95% (1.06)	95.78% (1.00)
**F-Measure**	0.868 (0.06)	0.935 (0.05)	0.959 (0.03)	0.957 (0.02)
**Kappa statistic**	0.8651 (0.01)	0.9336 (0.01)	0.9586 (0.01)	0.9569 (0.01)
**Mean absolute error**	0.0056 (0.01)	0.0026 (0.01)	0.0086 (0.01)	0.0051 (0.01)
**Root mean squared error**	0.0662 (0.01)	0.0439 (0.01)	0.0478 (0.01)	0.0392 (0.01)
